# Expression patterns of FSHD-causing DUX4 and myogenic transcription factors PAX3 and PAX7 are spatially distinct in differentiating human stem cell cultures

**DOI:** 10.1186/s13395-017-0130-1

**Published:** 2017-06-21

**Authors:** Premi Haynes, Kelly Kernan, Suk-Lin Zhou, Daniel G. Miller

**Affiliations:** Division of Genetic Medicine, Department of Pediatrics, University of Washington, Campus Box 358056, 850 Republican Street, Room N416, Seattle, WA 98109 USA

**Keywords:** Facioscapulohumeral, FSHD, DUX4, PAX7, PAX3, Myogenesis, Isogenic, Stem cells

## Abstract

**Background:**

Facioscapulohumeral muscular dystrophy (FSHD) is most commonly inherited in an autosomal dominant pattern and caused by the abnormal expression of DUX4 in skeletal muscle. The DUX4 transcription factor has DNA binding domains similar to several paired class homeotic transcription factors, but only myogenic factors PAX3 and PAX7 rescue cell viability when co-expressed with DUX4 in mouse myoblasts. This observation suggests competition for DNA binding sites in satellite cells might limit muscle repair and may be one aspect of DUX4-associated myotoxicity. The competition hypothesis requires that DUX4 and PAX3/7 be expressed in the same cells at some point during development or in adult tissues. We modeled myogenesis using human isogenic iPS and ES cells and examined expression patterns of DUX4, PAX3, and PAX7 to determine if conditions that promote PAX3 and PAX7 expression in cell culture also promote DUX4 expression in the same cells.

**Methods:**

Isogenic iPSCs were generated from human fibroblasts of two FSHD-affected individuals with somatic mosaicism. Clones containing the shortened FSHD-causing D4Z4 array or the long non-pathogenic array were isolated from the same individuals. We also examined myogenesis in commercially available hES cell lines derived from FSHD-affected and non-affected embryos. DUX4, PAX3, and PAX7 messenger RNAs (mRNAs) were quantified during a 40-day differentiation protocol, and antibodies were used to identify cell types in different stages of differentiation to determine if DUX4 and PAX3 or PAX7 are present in the same cells.

**Results:**

Human iPS and ES cells differentiated into skeletal myocytes as evidenced by Titin positive multinucleated fibers appearing toward the end of a 40-day differentiation protocol. PAX3 and PAX7 were expressed at similar times during differentiation, and DUX4 positive nuclei were seen at terminal stages of differentiation in cells containing the short D4Z4 arrays. Nuclei that expressed both DUX4 and PAX3, or DUX4 and PAX7 were not observed after examining immunostained nuclei at five different time points during myogenic differentiation of pluripotent cells.

**Conclusions:**

We conclude that DUX4, PAX3, and PAX7 have distinct expression patterns during myogenic differentiation of stem cells. Our findings are consistent with the hypothesis that muscle damage in FSHD is due to DUX4-mediated toxicity causing destruction of terminally differentiated myofibers. While these studies examine DUX4, PAX3, and PAX7 expression patterns during stem cell myogenesis, they should not be generalized to tissue repair in adult muscle tissue.

**Electronic supplementary material:**

The online version of this article (doi:10.1186/s13395-017-0130-1) contains supplementary material, which is available to authorized users.

## Background

Facioscapulohumeral muscular dystrophy (FSHD) is a myopathy most commonly inherited in an autosomal dominant pattern with a prevalence of 12 in 100,000 individuals [1]. Affected individuals have progressive and often asymmetric muscle weakness that begins in the face and shoulder region but ultimately affects most skeletal muscles [2]. Benign retinal vascular tortuosity is common in FSHD, and a small percentage of FSHD-affected individuals also have vision [3, 4] and hearing [5] loss. FSHD is most often caused by the contraction of D4Z4 arrays in the subtelomeric region of chromosome 4 leading to epigenetic de-repression of DUX4 [6–10], a homeotic transcription factor normally expressed in the testes [10], and thymus [11]. Both forced [12–14] and endogenous [15] DUX4 expression lead to cell death in a variety of cells including cultured human myocytes.

The DUX4 retrogene is unique to the primate lineage [16, 17], but arrayed retro-orthologs exist in other species including mouse [16]. Endogenous expression of DUX4 deregulates over ~500 different human transcripts while overexpression of human DUX4 in mouse cells deregulates ~183 mouse transcripts with an overlap of only 43 genes [18]. Only 6% of the human genes upregulated by DUX4 are also upregulated by forced mouse Dux expression in human cells [19]. This vast difference in species-specific target-gene activation makes it difficult to interpret experiments involving either expression of human DUX4 in mouse cells or mouse Dux in human cells. Furthermore, transgenic mice carrying the human DUX4 gene lack myopathic changes suggesting that some features of FSHD may be secondary to the chromosomal context of the arrayed DUX4 gene and species-specific differences in DUX4 targets [20, 21]. Studying human DUX4 in human muscle tissue, or in cultured human myocytes, can also be difficult due to low, infrequent DUX4 expression [10, 15, 22]. DUX4 mRNA can be detected in muscle biopsies from FSHD-affected individuals [10], but protein staining has been difficult to demonstrate. Alternatively, DUX4 expression can be observed in cultured human myocytes but DUX4 protein from endogenous DUX4 genes in their arrayed context on chromosome 4 is present in 0.5 to 4.3% of cultured differentiated FSHD myocytes with variability seen between individuals [15, 22].

Clues to the normal function of DUX4 may be present in the sequence homology of the protein where DUX4 DNA binding domains are most similar to the DNA binding domains of paired class homeotic transcription factors. PAX3 and PAX7 have been shown to be important regulators of myogenesis [23–25], so competition for similar DNA binding sites has been proposed as a possible mechanism of the muscle specific pathogenesis of aberrant DUX4 expression [14, 26]. Mutation of PAX3 can lead to craniofacial abnormalities and hearing loss thought to be caused by dysfunctional neural crest cell migration [27–29], and neural crest cell dysfunction may also explain the visual and auditory pathologies associated with FSHD although this has not been demonstrated. PAX7 is expressed in satellite stem cells that play a critical role in proliferation and differentiation during myofiber regeneration and repair [24]. Overexpression of PAX3 or PAX7 in mouse myoblasts (but not overexpression of other paired class homeotic transcription factors) has been shown to reduce DUX4 toxicity by competing for DNA binding sites and has been proposed as a possible mechanism of FSHD pathology [14]. Further support for this model comes from the observation that satellite cell proliferation is inhibited during muscle regeneration in mice containing DUX4 transgenes [26]. An important tenant of the competition hypothesis is the requirement that DUX4 and PAX3 or PAX7 be expressed in the same cell.

Homeotic transcription factors are expressed early in development where they are involved in cell fate decisions. Thus, human stem cell models of myogenesis will be important for establishing DUX4 expression patterns and determining if DUX4 and PAX3 or PAX7 are co-expressed. Two studies [30, 31] recently describe independently developed protocols to differentiate human stem cells into skeletal myocytes. While DUX4 expression has been followed previously in model systems utilizing ES cells from FSHD-affected embryos and iPS cells [10, 31], cellular localization of DUX4, PAX3, and PAX7 was not examined. In addition, isogenic stem cell lines with and without contracted D4Z4 arrays would be valuable for comparing FSHD-related cell culture phenotypes. Somatic mosaicism of D4Z4 array contractions has been reported in several individuals with FSHD and has been identified in as many as 40% of de novo cases [32]. Cells from these individuals present the opportunity to create isogenic pairs of cell lines differing only in the length of the D4Z4 array [18].

We obtained fibroblasts from two FSHD-affected individuals with somatic mosaicism and generated iPS cells. IPS cell clones containing either long or short D4Z4 arrays were isolated from the same individual, expanded, and characterized. Using a recently published differentiation protocol [30], we identified myogenic cell populations that were PAX3, PAX7, or Titin positive representing early, middle, and late stages of myogenesis. We examined PAX3- and PAX7-expressing cell populations for evidence of DUX4 co-expression by immunostaining isogenic human cells with DUX4 expressed from its arrayed context and location at the chromosome 4 subtelomere.

## Methods

### Ethics statement

Fibroblasts previously obtained from de-identified human biopsies from individuals with FSHD were provided by the Fields Center for FSHD Research biorepository and utilized in this study to create human iPS cell clones. Commercially available human ES cell line WA14 [33] was acquired from WiCell Research Institute at University of Wisconsin, Madison, WI, and the FSHD-affected ES cell line GENEA049 [34] was obtained from Genea Biocells, Sydney, New South Wales, Australia. This study was performed in accordance and approval of the University of Washington Institutional Review Board.

### Generation of isogenic iPS cell clones from individuals with FSHD

Fibroblasts were obtained from two individuals affected by FSHD who were identified to have somatic mosaicism through genetic testing for the D4Z4 array sizes on chromosome 4. Human iPS cell clones were generated using the protocol of Takahashi et al. [35]. Individual clones were isolated and array sizes measured by hybridizing the p13E11 probe [6] to Southern blots of DNA fragments separated by pulse field gel electrophoresis.

### Southern blots to identify array sizes

D4Z4 array lengths were measured in human iPS and ES cells according to the protocol posted on the website for the Fields Center for FSHD Research (https://www.urmc.rochester.edu/fields-center/research-info/protocols.aspx). Briefly, cells were embedded in agarose plugs and incubated in pronase and sarcosyl to lyse cells and expose the DNA. The DNA was digested within the agarose plugs by treatment with *Eco*RI/*Hind*III, *Eco*R1/*Bln*I, or *Eco*R1/*Xap*I, and DNA fragments were separated using pulse field gel electrophoresis. Alternatively, individual clones isolated from each mosaic individual were screened for evidence of a shortened D4Z4 array using standard gel electrophoresis of *Eco*RI digested genomic DNA isolated from cell pellets. The DNA fragments were transferred to nylon membranes (Hybond N+) and hybridized with a P^32^-labeled p13E-11 probe [6] that is complimentary to the proximal end of the *Eco*RI fragment containing D4Z4 arrays from both chromosomes 4 and 10. D4Z4 arrays on chromosomes 4 and 10 are distinguished by their sensitivity to digestion with *Bln*I (chromosome 10) and *Xap*I (chromosome 4). D4Z4 *Eco*R1 fragment sizes ≥42 kb are generally non-pathogenic, and *Eco*R1 D4Z4 fragments ≤42 kb present on permissive 4qA-type haplotypes are generally pathogenic for FSHD [36].

### Myogenic differentiation of human iPS and ES cell lines in cell culture

Human stem cells were cultured on irradiated mouse embryonic fibroblasts in media containing F12/DMEM (Life Technologies), 20% Knock out serum replacer (Life Technologies), 1× penicillin/streptomycin (Life Technologies), 1% 100 mM sodium pyruvate (Life Technologies), 1× non-essential amino acid (Life Technologies), 0.1% 0.1 M β-mercaptoethanol (Sigma), and 2 ng/mL FGF-2 (Life Technologies). Stem cell colonies were separated from irradiated mouse embryonic fibroblast layers using dispase (Life Technologies) and seeded to matrigel-coated dishes in mTESR1 media (Stemcell Technologies). The next day, single cell suspensions were generated by treating the adherent cells with TrypLE (Life Technologies) and the cells were seeded to matrigel-coated dishes (5000 cells/cm^2^) containing mTESR1 and cultured at 37 °C in 5% oxygen and 5% CO_2_. The cells were maintained in serum-free base media containing DMEM/F12 with 1% ITS and 0.2% penicillin/streptomycin from day 3 to 6. Medium on days 3–4 also contained 3 μM CHIRON99021 (Stemgent) and 0.5 μM LDN193189 (Stemgent). Medium on days 5–6 also contained 3 μM CHIRON99021 and 0.5 μM LDN193189 and 20 ng/mL FGF-2. On day 7 and through day 30, the base media was changed to DMEM/F12+ 15% KOSR+ 0.2% penicillin/streptomycin with 10 ng/mL HGF (Thermofisher), 2 ng/mL IGF-1 (R &D systems), 20 ng/mL FGF-2, and 0.5 μM LDN193189 added for days 7–8, and 2 ng/mL IGF-1 added for days 9–12. For the remaining differentiation (days 13–30), cells were cultured with 10 ng/mL HGF and 2 ng/mL IGF-1 and moved to normal oxygen concentrations and 5% CO_2_. Cells were detached with TrypLE on day 31 and cultured by reseeding every 10 days in DMEM/F12+ 1% penicillin/streptomycin+ 1% ITS (Life Technologies) + 1% N2 supplement (Life Technologies). This myogenic differentiation protocol was adapted and modified from a previously published protocol [30, 37].

### Immunofluorescence staining and microscopy

Immunofluorescence staining was performed on differentiating cell cultures at five different time points (D0, D7, D21, D30, and D40). Cells were washed with PBS and fixed in 4% formalin solution for 15 min at room temperature followed by a wash in PBS containing 0.005% Triton X-100 (PBS-T). Cells were permeabilized by treatment with PBS containing 0.5% Triton X-100 for 10 min, washed in PBS-T, and incubated with primary antibodies and 500 nM DAPI overnight. The next day, the cells were washed three times with PBS-T and incubated with fluorescent-conjugated Alexa secondary antibodies for 2 h at room temperature, followed by a PBS-T wash. The primary antibodies used in this study were anti-PAX3 mouse monoclonal antibody (1:200 dilution, DSHB), anti-PAX7 mouse monoclonal antibody (1:200 dilution, DSHB), anti-Myogenin mouse monoclonal antibody (1:250 dilution, DSHB), anti-Titin mouse monoclonal antibody (1:200 dilution, DSHB), and anti-DUX4 E5-5 rabbit polyclonal antibody (1:1000 dilution, Abcam). Secondary antibodies included Alexa 448, 594 and 647 anti-rabbit or anti mouse at 1:5000 dilution (Life Technologies). Images were captured with a Nikon TiE inverted widefield fluorescence microscope and processed using the elements software at the University of Washington’s Lynn and Mike Garvey Cell Imaging Laboratory.

### RNA extraction and qRT-PCR analysis

RNA was extracted from cultured cells at five different time points (D0, D7, D21, D30, and D40). One milliliter of TRIzol Reagent (Life Technologies) was added to a 3.5-cm-diameter dish followed by a 10 min incubation at room temperature. Two hundred microliters of chloroform was added to the TRIzol, mixed by agitation, and organic and aqueous phases separated by centrifugation. RNA was precipitated from the aqueous phase by adding 500 μL of isopropanol and centrifugation. RNA pellets were washed with 70% EtOH, and re-suspended with 50 μL of water. Five units of DNAseI (New England BioLabs) was added to 10 μg of RNA from each sample and incubated at 37 °C for 15 min. RNA from these treated samples was diluted in 500 μL RPE buffer followed by binding and elution from RNeasy columns (Qiagen RNeasy Mini Kit). Two micrograms of RNA was reverse transcribed to complementary DNA (cDNA) using oligo-dT primers and SuperScript III Reverse Transcriptase (Thermo Fisher); reactions without enzyme were also performed as controls. All reactions were incubated at 65 °C for 5 min, 50 °C for 50 min, and 85 °C for 5 min. Samples were then diluted 1:4 with RNAse free water, for use in RT-PCR analysis for GAPDH (F–GTGAAGGTCGGAGTCAAC, R-TGAGGTCAATGAAGGGGTC), PAX3 (F–TGCCGTCAGTGAGTTCCATCAGC, R–GCTAAACCAGACCTGTACTCGGGC), PAX7 (F—AACCACATCCGCCACAAGATA, R—GCCTGGGTTTTCCCTCTTGTA), DUX4 (F—GGCCCGGTGAGAGACTCCACA, R—CCAGGAGATGTAACTCTAATCCAGGTTTGC), and DUX4 Long Last Partial (F—CTCCCGACACCCTCGGACAGCAC, R—TCCAGGTTTGCCTAGACAGCGTC) using the Roche Fast Start Universal SYBR Mastermix with ROX (Roche). Polymerase chain reactions were carried out on a ABI 7300 machine (Applied Biosystems) programed to incubate samples at 50 °C for 2 min, 95 °C for 10 min, and then 40 cycles of 95 °C for 15 s, 60 °C for 30 s, and 72 °C for 30 s. Analyses of all samples were performed in replicates.

## Results

### Generation and characterization of isogenic pairs of hiPS cell clones derived from FSHD-affected individuals with mosaicism

**Fig. 1:**
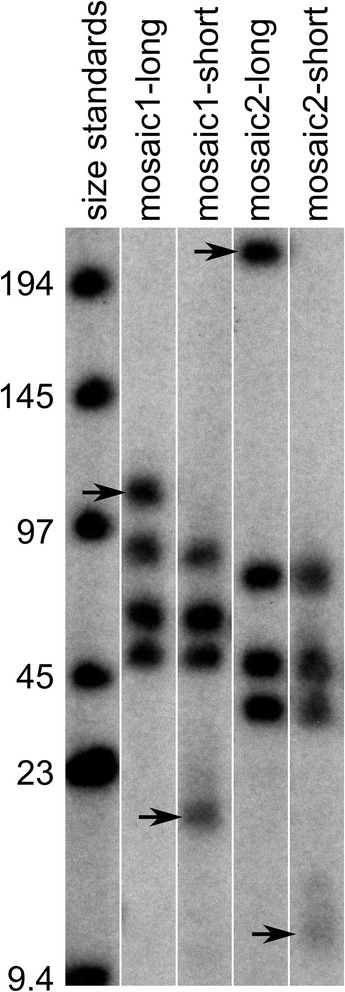
Southern blot of genomic DNA from isogenic pairs of stem cell clones derived from FSHD-affected individuals with mosaicism. Genomic DNA from hiPS cell clones was digested with *Eco*RI and *Hin*DIII restriction endonucleases and separated by size on an agarose gel using pulsed field gel electrophoresis. A Southern blot of this gel was probed with P^32^-labeled p13E-11 fragments and exposed on autoradiography film. The *bands* shown in each lane correspond to maternal and paternal D4Z4 arrays on chromosomes 4 and 10 (4 arrays/lane). *Lanes 2* and *3* show D4Z4 array sizes present in hiPSC clones derived from mosaic1 with a 107 kb chromosome 4 array in the “long” clone and the same array reduced to ~19 kb size in the “short” clone (indicated by *arrows*). *Lanes 3* and *4* show D4Z4 array sizes from hiPSC clones derived from mosaic2 with a 220 kb chromosome 4 array in the “long” clone and the same array reduced to ~15 kb in the “short” clone from the same person (indicated by *arrows*). DNA size markers are shown in *lane 1*. Chromosome 4-type and 10-type arrays were distinguished by further digestion with *Xap*I to remove chromosome 4-type arrays or *Bln*I to remove chromosome 10-type arrays. Additional blots were probed with “A” and “B” probes recognizing the distal end of the arrays to confirm that the chromosome 4 arrays varying in size are permissive A-type arrays generally known to produce FSHD when <11 units long

Somatic mosaicism for different D4Z4 array lengths has been observed in 40% of FSHD presenting de novo [32]. To focus on FSHD-associated differences in gene expression, and DUX4 expression during stem cell differentiation, we generated isogenic iPS cell clones from the biopsies of FSHD-affected individuals with somatic mosaicism. Mosaic populations of fibroblasts were transduced with retroviruses encoding OCT4, SOX2, KLF4, and cMYC, and iPS cells isolated following standard protocols [35]. IPS cell clones were screened by probing Southern blots of genomic DNA with p13E11 and isolating clones containing either short (FSHD-causing) or long (non-pathogenic) arrays [38]. We selected one pair of isogenic clones from two unrelated FSHD-affected individuals for further study (four clones total). Pathogenic array sizes were 19 and 15 kb corresponding to four and three D4Z4 units respectively (Table 1 and Fig. 1). We also included previously characterized human ES cell lines isolated from both affected (hESC-FSHD) [34] and unaffected (hESC-Cntrl) [33] embryos.

**Table 1:** Human iPS and ES cell clone properties and D4Z4 array sizes

Cells	Allele^a^	Array status	^b^Haplotype	^d^D4Z4 (kb)
hESC-Cntrl	1	Non-pathogenic	^c^4qA161	91
	2	Non-pathogenic	4qB168	44
	3	Non-pathogenic	4qB168	67
hESC-FSHD	1	Non-pathogenic	4qA	135
	2	Pathogenic	4qA	22
hiPSC-mosaic1-long	1	Non-pathogenic	4qA161	107
	2	Non-pathogenic	4qB163	89
hiPSC-mosaic1-short	1	Pathogenic	4qA161	19
	2	Non-pathogenic	4qB163	89
hiPSC-mosaic2-long	1	Non-pathogenic	4qA161	220
	2	Non-pathogenic	4qA	70
hiPSC-mosaic2-short	1	Pathogenic	4qA161	15
	2	Non-pathogenic	4qA	70

^a^Chromosome 4 allele

^b^A (permissive) or B (non-permissive) and SSLP length

^c^A161 allele is a hybrid of 4 and 10 type arrays

^d^ *Eco*RI fragment size for D4Z4 array on chromosome 4 in kilobase pairs

### Stem cell clones differentiate into Titin positive multinucleate skeletal myocytes after progressing through PAX3 and PAX7 positive stages

**Fig. 2:**
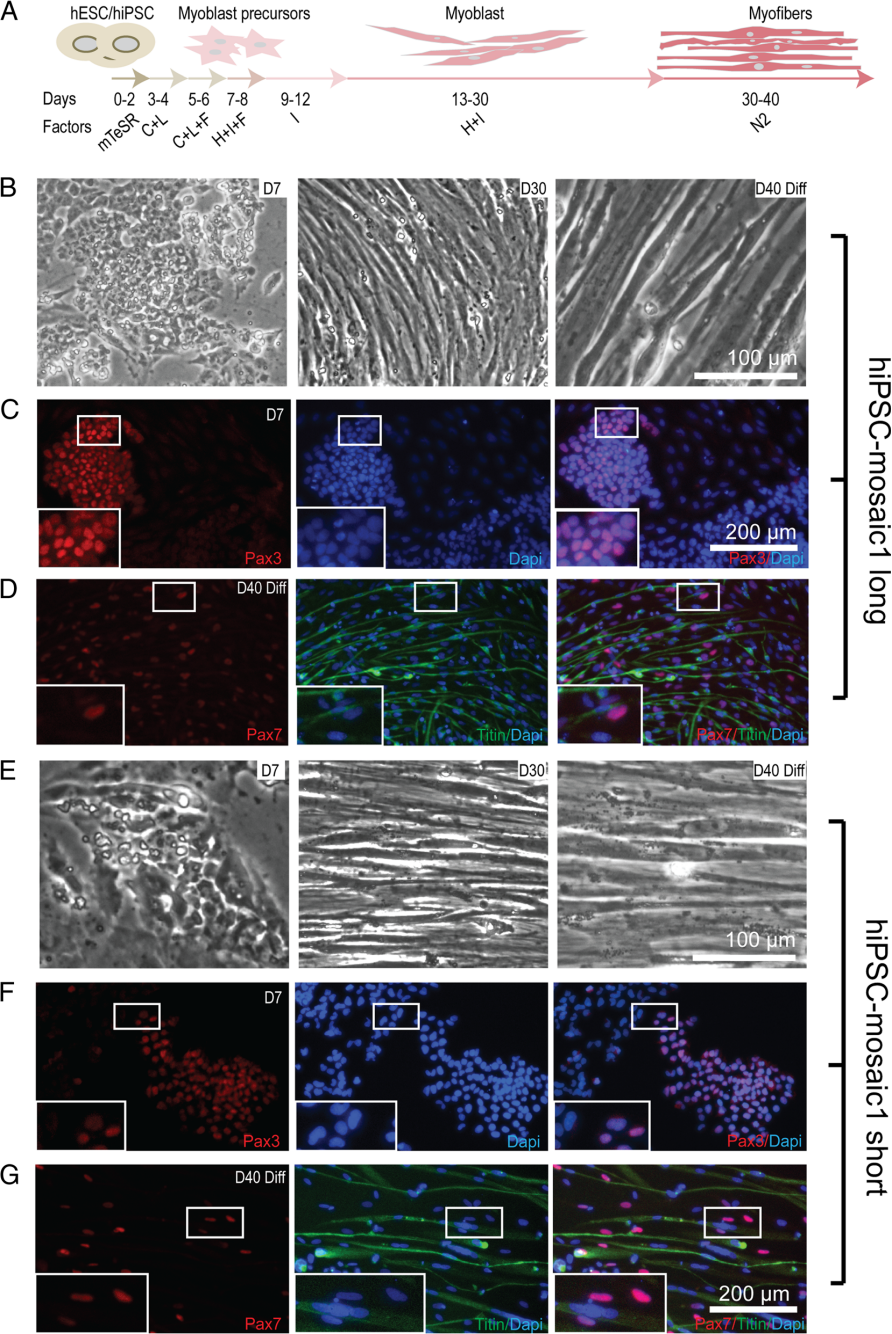
Human ES cells and iPS cell clones differentiate into skeletal myocytes with progressive expression of myogenic markers characteristic of early, middle, and late stages of myogenesis. **a** Schematic of the differentiation protocol. *C* = CHIRON99021, *L* = LDN193189, *F* = FGF2, *H* = HGF, *I* = IGF, *N2* = neuronal supplement. **b**–**d** Images of isogenic clone hiPSC-mosaic1 with the long D4Z4 array. **e**–**g** Images of hiPSC-mosaic 1 with the short D4Z4 array. **b, e** Bright field images of cells at the various stages shown in **a**. Cell morphology proceeds from small stem-like cells (D7) to spindle-shaped elongated cells more characteristic of myoblasts (D30) to multinucleate elongated fibers on D40. **c**, **f** Immunofluorescence images of PAX3-stained cells early in the protocol (D7) that progress to differentiated myocytes with **d, g** immunoreactivity for PAX7and Titin. *Insets* show magnified view of cells within the *white boxes*

Efficient differentiation of stem cells to skeletal myocytes without forced expression of myogenic transcription factors has only recently been successful [30, 31, 37]. We adapted a modified version of a published protocol [30, 37] to differentiate stem cells into myocytes. Cell morphology gradually changed over the 40-day protocol from clusters of small stem-like cells with high nuclear to cytoplasmic ratios (D0-D7) to elongated and aligned myoblast-like cells (D30) that became longer and multinucleated by D40 (Fig. 2a, b, e). Antibodies reactive to myogenic markers that are characteristic of early (PAX3), middle (PAX7), and late (Titin) stages of myogenesis were used to follow myogenic progression during the 40-day protocol (Fig. 2c, d, f, g, Additional files 1 and 2: Figures S1 and S2). We confirmed that we could obtain PAX3+ and PAX7+ myocytes and terminally differentiated myotubes using this protocol; however, we found that clear-ordered sequential gene expression shown in the original report was cell line dependent and the efficiency of myocyte production seemed to vary between different starting cell lines [30, 37]. We also found that human ES and IPS cells with short and long D4Z4 arrays differentiate into myocytes without obvious differences in their ability to produce PAX3+ and PAX7+ cells. The cultures generally progressed stepwise beginning with no detectable PAX3 or PAX7 expression, to expression of PAX3 seen most abundantly early in the protocol, to cells expressing PAX7, and to elongated multinucleate post-mitotic cells expressing markers of terminally differentiated myocytes broadly paralleling steps previously observed in vivo [25].

### PAX3 and PAX7 transcripts are present, and DUX4 is expressed in cells containing short D4Z4 arrays during myogenic differentiation

**Fig. 3:**
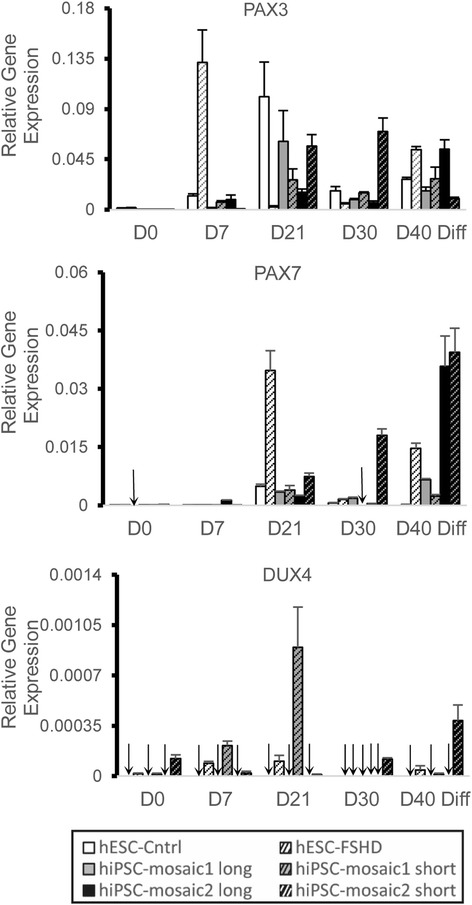
RNA expression profiles of myogenic regulators PAX3 and PAX7 and DUX4 expression in cultures of human ES cells and iPS cell clones during myogenic differentiation. RNA was isolated at five time points (D0, D7, D21, D30, and D40) representing early, middle, and late stages of myogenesis. Quantitative PCR of PAX3, PAX7, and DUX4 RNA transcripts was performed at each time point, and values normalized to GAPDH RNA levels in the same preparations. Oligonucleotide primers designed to amplify the 3′ end of the DUX4 gene from the terminal D4Z4 unit on chromosome 4 were used to quantify DUX4 transcripts. Undetected RNA transcripts are marked with an *arrow*

PAX3 and PAX7 expression was measured by quantitative PCR in all six cell lines at five separate time points during the 40-day differentiation protocol. Both PAX3 and PAX7 expression were undetectable at D0 but began increasing around D7 (Fig. 3a). In general, PAX3 expression peaked around D21 and decreased in the latter half of the protocol (Fig. 3a). PAX7 expression started peaking at D21 (Fig. 3b) and was highly expressed at D40 consistent with the temporal hierarchy of its expression in vivo [25, 39]. These results indicate that PAX3 transcripts are expressed in early stages and PAX7 transcripts are expressed in middle to late stages of myogenic differentiation and that both human ES and iPS cells from different individuals progress through similar myogenic differentiation sequences.

DUX4 transcripts have been reported in thymus tissue and testis from unaffected individuals, and in cultured skeletal myocytes and muscle tissue from FSHD-affected individuals [10, 11, 15]. DUX4 transcription is normally silenced in differentiated cells where D4Z4 arrays show CpG methylation and histone modifications characteristic of repressive chromatin states [10, 40]. We used quantitative PCR to follow DUX4 messenger RNA (mRNA) transcripts during myogenic differentiation to determine the gene expression profile of DUX4 in cells from FSHD-affected and unaffected individuals. DUX4 gene expression was detected in iPS and ES cells that contain short pathogenic D4Z4 arrays with the highest expression levels in iPS cells containing short D4Z4 arrays (Fig. 3c). While DUX4 transcripts were detected throughout the 40-day differentiation protocol in cells containing short D4Z4 arrays, DUX4 mRNA was undetectable in differentiated ES and iPS cells containing long non-pathogenic D4Z4 arrays consistent with the previously suggested role in early development but pathogenic expression in terminally differentiated myocytes.

### DUX4, PAX3, and PAX7 are not observed together in the same cell during myogenic differentiation

**Fig. 4:**
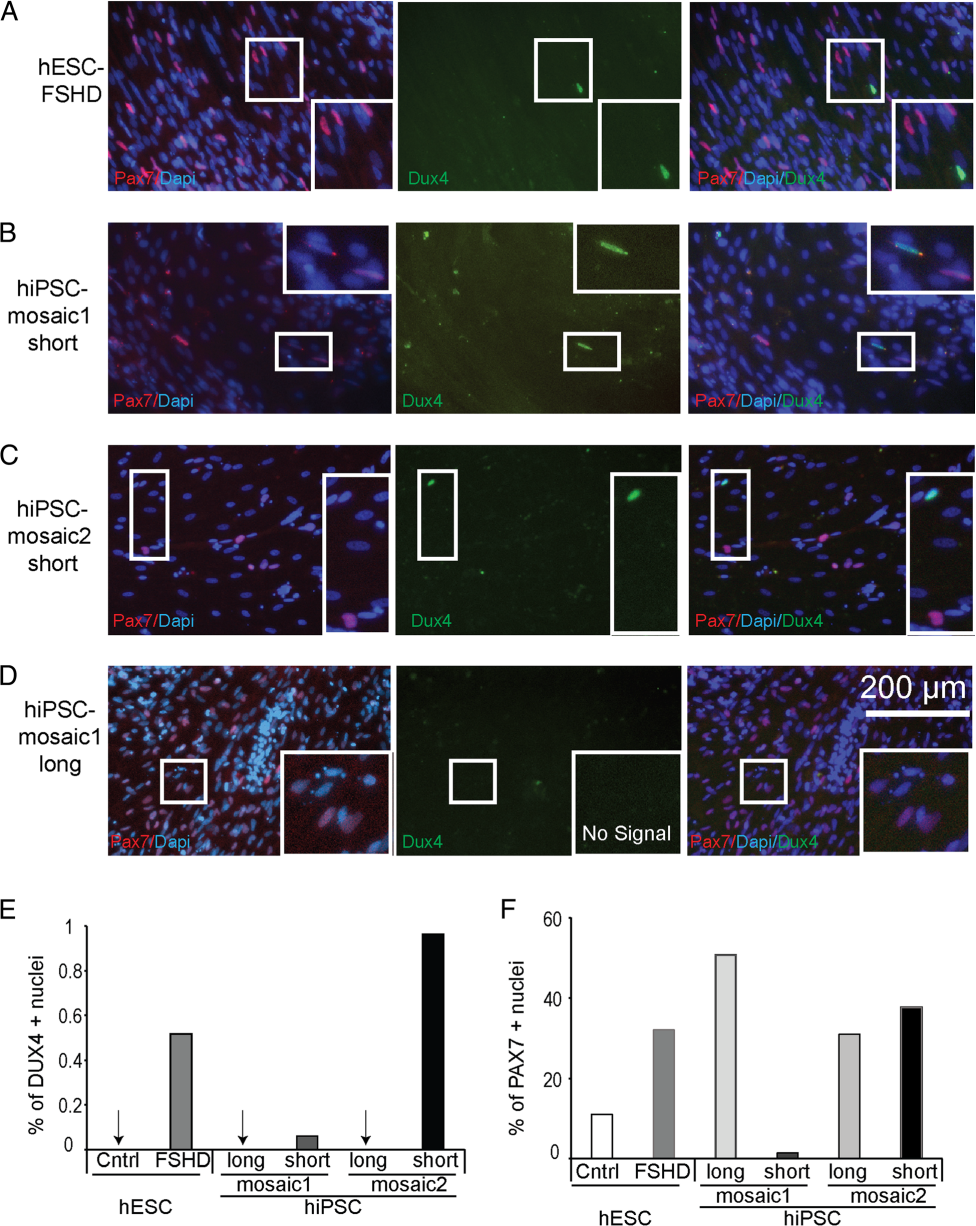
DUX4 and PAX7 are expressed in distinct cell types during myogenic differentiation. Representative images of DUX4 positive cells from D40 of the differentiation protocol stained with antibodies to both PAX7 and DUX4. **a** hESC-FSHD, **b** hiPSC-mosaic1-short, **c** hiPSC-mosaic2-short, and **d** hiPSC-mosaic1-long showing PAX7 positive nuclei but no DUX4 signal. Bar graph quantifying the **e** percentage of DUX4 positive nuclei and **f** percentage of PAX7 positive nuclei for the given number of DAPI-stained nuclei

RNA analysis of differentiating cultures containing mixtures of cell types provides a limited view of potential interaction between DUX4 and PAX3 or PAX7. Therefore, we identified and localized DUX4, PAX3, and PAX7 protein by immunostaining cells in differentiating cultures with antibodies recognizing each transcription factor. All proteins were localized to the nucleus and present in a fraction of the cultured cells. DUX4 protein was present in a small fraction of cells from all cultures containing short D4Z4 arrays and was most consistently detected in terminally differentiated cells present on D40 of the protocol. All fields containing abundant PAX3 staining cells did not show any cells that stained for DUX4 (Table 2). In several microscopic fields, we identified DUX4 and PAX7 positive nuclei present in different cells confirming that their expression could be temporally related as the qPCR results suggested (Fig. 3) but the presence of DUX4 and PAX7 in the same cell was never observed (Fig. 4a–c, Additional files 3, 4, 5, 6, 7 and 8: Figures S3–S8, Table 2). PAX7 positive nuclei were observed in differentiated iPS cell cultures containing long D4Z4 arrays, but DUX4 was undetectable in these cells consistent with the RNA analysis (Fig. 3). The percentage of DUX4 positive nuclei ranged from 0.1 to ~1%, and PAX7 was present in 1–55% of cells depending on the cell line (Fig. 4e, f, Table 2).

**Table 2:** Frequency of PAX3/DUX4 and PAX7/DUX4 double positive nuclei in myogenic cultures

Cells	TF	^a^Number of positive cells at indicated time				Observed double + frequency (D40)		Expected double + frequency (D40)	
		D7	D21	D30	D40	PAX3/DUX4	PAX7/DUX4	PAX7/DUX4	^#^ *p* value
hESC-Cntrl	*PAX7*	0	56	20	54	<1/150	<1/54	0	na
	*PAX3*	22	68	60	0				
	*DUX4*	0	0	0	0				
	*Dapi*	nd	nd	nd	491				
hESC-FSHD	*PAX7*	0	325	289	373	<1/82	<1/373	1/605	.098
	*PAX3*	52	20	10	0				
	*DUX4*	0	0	0	6				
	*Dapi*	nd	nd	nd	1164				
hiPSC-mosaic1-long	*PAX7*	0	360	320	1456	<1/565	<1/1456	0	na
	*PAX3*	75	458	32	0				
	*DUX4*	0	0	0	0				
	*Dapi*	nd	nd	nd	2873				
hiPSC-mosaic1-short	*PAX7*	0	40	115	70	<1/566	<1/70	1/114,753	.958
	*PAX3*	84	359	123	0				
	*DUX4*	0	0	0	3				
	*Dapi*	nd	nd	nd	4909				
hiPSC-mosaic2-long	*PAX7*	0	8	45	127	<1/162	<1/127	0	na
	*PAX3*	110	35	17	0				
	*DUX4*	0	0	0	0				
	*Dapi*	nd	nd	nd	410				
hiPSC-mosaic2-short	*PAX7*	0	19	153	1678	<1/102	<1/1678	1/276	<.0001
	*PAX3*	93	5	4	0				
	*DUX4*	0	0	0	43				
	*Dapi*	nd	nd	nd	4460				

*nd* not done, *na* not applicable, *TF* transcription factor

^a^D0 cells were negative for DUX4, PAX3, and PAX7 staining

^#^D40 *p* values are from a table of single tailed values (Fisher’s exact test)

When comparing expected PAX7/DUX4 double positive cell frequencies calculated by multiplying the frequency of PAX7 and DUX4 positive nuclei on D40 (Fig. 4e, f) to the observed double positive frequencies (<1 over the number of D40 PAX7 positive cells counted), we found that observed frequencies were statistically less than expected frequencies in hiPSC-mosaic2-short (Table 2, *p* < .0001) and approached statistical significance in the embryonic stem cells hESC-FSHD (Table 2, *p* < .098). DUX4 positive nuclei were infrequent making the number of scored DUX4+ nuclei small, but the lack of DUX4 expression in the significant number of both PAX7 and PAX3 cells counted in these differentiating cultures demonstrates that whether observed or predicted DUX4/PAX7 double positive cells are extremely rare.

Combining the frequency of DUX4/PAX7 co-expression in hESC-FSHD, hiPSC-masaic1-short, and hiPSC-mosaic2-short (three independent lines), the average frequency of DUX4/PAX7 positive nuclei is <1/500 with a standard deviation of 0.0021 (Table 2). The same calculation for PAX3-stained cultures gives an average frequency of DUX4/PAX3 positive cells of <1/127 with a standard deviation of 0.0055. These observations suggest that DUX4 and PAX7 or DUX4 and PAX3 are rarely if ever co-expressed when pluripotent stem cells are induced to differentiate to myocytes.

## Discussion

The similarity of DUX4 to other homeotic transcription factors suggests that events occurring during early development may play a role in FSHD pathology. The lack of animal models that recapitulate the disease phenotype precludes the study of DUX4 expression in early development [41]. While the study of human development requires embryos and associated ethical implications, directed differentiation of pluripotent embryonic and induced pluripotent stem cells can give clues to the involvement of DUX4 with transcriptional events that are important for cell identity and tissue formation. Recently, several laboratories have developed protocols to differentiate stem cells into myocytes without forced expression of myogenic transcription factors or sorting for rare cells [30, 31, 37], and in this study, we were able to successfully generate PAX3+ and PAX7+, and terminally differentiated multinucleated myotubes using one published differentiation protocol [30] and study DUX4 expression relative to several myogenic transcription factors.

We extended the utility of modeling aspects of FSHD using stem cells by isolating isogenic pairs of stem cell clones from individuals exhibiting somatic mosaicism for the contracted FSHD-causing D4Z4 array. The paired clones of iPS cells generated for this study differ only in D4Z4 array length and will be useful for studying the effects of array length on myocyte formation and the analysis of previously described tissue culture phenotypes [31]. Our DUX4 expression analysis in differentiating cells supports the long-standing hypothesis that D4Z4 array length is the nidus for establishing a repressive chromatin state and further suggests that repressive chromatin is established early in human development during the formation of skeletal myocytes. Here, we demonstrate differential silencing of the D4Z4 array during myogenic differentiation indicating that this cell culture model faithfully recapitulates a central feature of FSHD. The isogenic stem cell pairs described here will be useful for determining the mechanism of array silencing, testing hypotheses of disease mechanism, and evaluating therapeutic approaches for FSHD treatment. We demonstrated the utility of this model system by investigating whether PAX3 or PAX7 positive cells co-express DUX4 under these culture conditions, a necessary feature of competition between these transcription factors and the basis of one theory for DUX4 pathogenicity [14, 26].

We found that PAX3 and PAX7 expression is readily detected in differentiating stem cell cultures but we were unable to show that DUX4 was present in cells expressing either PAX3 or PAX7. We emphasize that there are limitations to this study and that the absence of PAX3/PAX7/DUX4 co-expression in the culture conditions used here does not necessarily mean that DUX4 is never co-expressed with PAX3 or PAX7 at some point during human development, nor does this study rule out the possibility that DUX4 could compete with PAX3 or PAX7 during regeneration of adult tissues. Our study suggests that conditions that promote expression of PAX3 or PAX7 in myogenic cells do not also promote expression of DUX4 in the same cells. An additional caveat is the possibility that DUX4 expression reduces expression of PAX3 or PAX7 making their expression mutually exclusive but preserving the possibility that they may transiently compete for binding sites [12].

The absence of DUX4 expression in early myogenic cell types is consistent with the observation that stem cell cultures produce myocytes regardless of whether the stem cells were derived from unaffected or FSHD-affected individuals and the observation that most children with FSHD have normal muscle strength without delayed motor developmental milestones. These observations are also consistent with FSHD being a disease that primarily targets mature, terminally differentiated muscle fibers [10, 15, 22, 42].

The high frequency of somatic mosaicism in FSHD-affected individuals suggests that mosaic cell populations could be a source for isogenic non-pathogenic cells that could one day be used for transplantation. Efficient transplantation of myocytes derived from stem cells has been demonstrated [43], but these cells were produced by a different protocol that involved inducible PAX7 expression. Efficient transplantation of the PAX7 positive cell populations derived from this and other recently published protocols has not been demonstrated, so further investigation will be required before an isogenic transplantation approach can be tested.

## Conclusions

We show that DUX4, PAX3, and PAX7 have distinct spatial pattern of expression during differentiation of stem cells containing the short FSHD-causing D4Z4 array. The implication of this study is that these three transcription factors are unlikely to compete for the same genomic binding sites in differentiating stem cell cultures.

## Additional files

Additional file 1: Figure S1. Human iPS isogenic clones hiPSC-mosaic 2 with long and short D4Z4 array differentiate into skeletal myocytes with progressive expression of myogenic markers characteristic of early, middle, and late stages of myogenesis. Bright field images of A) hiPSC-mosaic2-long and D) hiPSC-mosaic2-short at the various stages of differentiation. Cell morphology proceeds from small stem-like cells (D7) to spindle-shaped elongated cells more characteristic of myoblasts (D30) to multinucleate elongated fibers on D40. Immunofluorescence images of B) hiPSC-mosaic2-long and E) hiPSC-mosaic2-short of PAX3-stained cells early in the protocol (D7) that progress to differentiated C) hiPSC-mosaic2-long and F) hiPSC-mosaic2-short myocytes with immunoreactivity for PAX7and Titin. Insets show magnified view of cells within the white boxes. (DOCX 2422 kb)

Additional file 2: Figure S2. Human ES cells differentiate into skeletal myocytes with progressive expression of myogenic markers characteristic of early, middle, and late stages of myogenesis. Bright field images of A) HESC-Cntrl and D) FSHD at the various stages of differentiation. Cell morphology proceeds from small stem-like cells (D7) to spindle-shaped elongated cells more characteristic of myoblasts (D30) to multinucleate elongated fibers on D40. Immunofluorescence images of B) HESC-Cntrl and E) FSHD of PAX3-stained cells early in the protocol (D7) that progress to differentiated C) HESC-Cntrl and F) FSHD myocytes with immunoreactivity for PAX7and Titin. Insets show magnified view of cells within the white boxes. (DOCX 2496 kb)

Additional file 3: Figure S3. DUX4 is not expressed in PAX7 positive myocytes in hiPSC-mosaic 1 with the long D4Z4 array. A, B, and C) Images of hiPSC-mosaic 1 long myocytes from D40 of the differentiation protocol stained with antibodies to both PAX7 and DUX4 and utilized to quantify the number of DUX4 and PAX7 positive cells. (DOCX 4880 kb)

Additional file 4: Figure S4. DUX4 and PAX7 are expressed in distinct cell types during myogenic differentiation of hiPSC-mosaic 1 with the short D4Z4 array. A, B and C) Images of hiPSC-mosaic 1 with the short D4Z4 array from D40 of the differentiation protocol stained with antibodies to both PAX7 and DUX4. Arrows indicate representative DUX4 positive nuclei counted. (DOCX 5052 kb)

Additional file 5: Figure S5. DUX4 is not expressed in PAX7 positive myocytes in hiPSC-mosaic 2 with the long D4Z4 array. A and B) Images of hiPSC-mosaic 2 long myocytes from D40 of the differentiation protocol stained with antibodies to both PAX7 and DUX4 and utilized to quantify the number of DUX4 and PAX7 positive cells. (DOCX 1755 kb)

Additional file 6: Figure S6. DUX4 and PAX7 are expressed in distinct cell types during myogenic differentiation of hiPSC-mosaic 2 with the short D4Z4 array. A, B, C, D and E) Images of hiPSC-mosaic 2 with the short D4Z4 array from D40 of the differentiation protocol stained with antibodies to both PAX7 and DUX4. Arrows indicate representative DUX4 positive nuclei counted. (DOCX 7778 kb)

Additional file 7: Figure S7. DUX4 is not expressed in PAX7 positive myocytes in control human ES cells. A and B) Images of hESC-cntrl myocytes from D40 of the differentiation protocol stained with antibodies to both PAX7 and DUX4 and utilized to quantify the number of DUX4 and PAX7 positive cells. (DOCX 1665 kb)

Additional file 8: Figure S8. DUX4 and PAX7 are expressed in distinct cell types during myogenic differentiation of human ES cells with FSHD. A, B, C, D, E and F) Images of hESC-FSHD from D40 of the differentiation protocol stained with antibodies to both PAX7 and DUX4. Arrows indicate representative DUX4 positive nuclei counted. (DOCX 6109 kb)

## Abbreviations

FSHD: Facioscapulohumeral muscular dystrophy
hESC: Human embryonic stem cells
hiPSC: Human induced pluripotent stem cell
