# Supplementary material for: Expression patterns of FSHD-causing DUX4 and myogenic transcription factors PAX3 and PAX7 are spatially distinct in differentiating human stem cell cultures
Source: Skelet Muscle. 2017 Jun 21;7:13. doi: 10.1186/s13395-017-0130-1 (PMC5480156; doi:10.1186/s13395-017-0130-1)
Supplement: Supplementary file 1 — Human iPS isogenic clones hiPSC-mosaic 2 with long and short D4Z4 array differentiate into skeletal myocytes with progressive expression of myogenic markers characteristic of early, middle, and late stages of myogenesis. Bright field images of A) hiPSC-mosaic2-long and D) hiPSC-mosaic2-short at the various stages of differentiation. Cell morphology proceeds from small stem-like cells (D7) to spindle-shaped elongated cells more characteristic of myoblasts (D30) to multinucleate elongated fibers on D40. Immunofluorescence images of B) hiPSC-mosaic2-long and E) hiPSC-mosaic2-short of PAX3-stained cells early in the protocol (D7) that progress to differentiated C) hiPSC-mosaic2-long and F) hiPSC-mosaic2-short myocytes with immunoreactivity for PAX7and Titin. Insets show magnified view of cells within the white boxes. (DOCX 2422 kb) [file 13395_2017_130_MOESM1_ESM.docx]

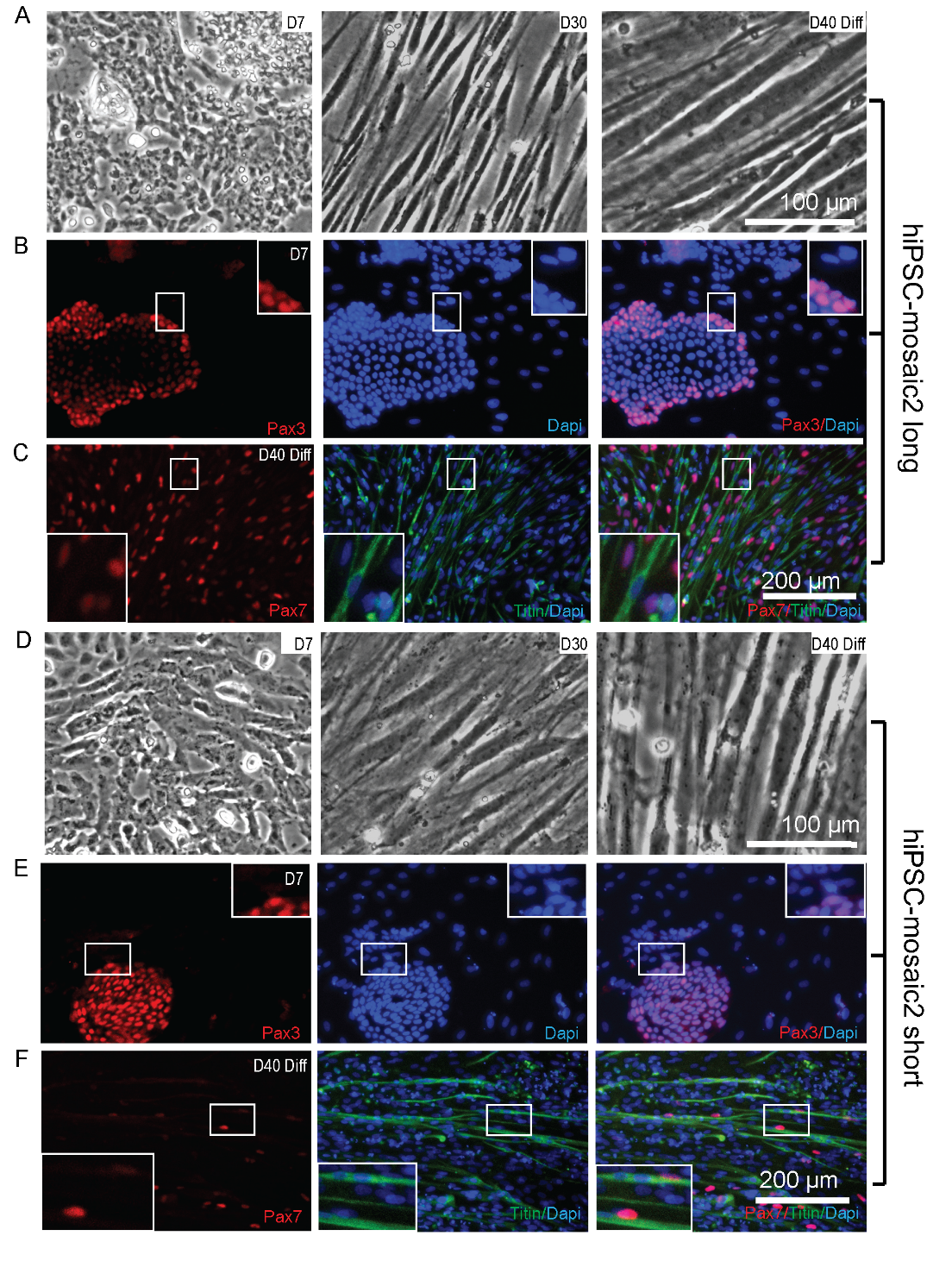


**Additional file 1: Figure S1. Human iPS isogenic clones hiPSC-mosaic 2 with long and short D4Z4 array differentiate into skeletal myocytes with progressive expression of myogenic markers characteristic of early, middle and late stages of myogenesis.** . Bright field images of A) hiPSC-mosaic 2 long and D) short at the various stages of differentiation. Cell morphology proceeds from small stem-like cells (D7) to spindle shaped elongated cells more characteristic of myoblasts (D30) to multinucleate elongated fibers on D40. Immunofluorescence images of B) hiPSC-mosaic 2 long and E) short of PAX3 stained cells early in the protocol (D7) that progress to differentiated C) hiPSC-mosaic 2 long and F) short myocytes with immunoreactivity for PAX7and Titin. Insets show magnified view of cells within the white boxes.
