# Supplementary material for: Expression patterns of FSHD-causing DUX4 and myogenic transcription factors PAX3 and PAX7 are spatially distinct in differentiating human stem cell cultures
Source: Skelet Muscle. 2017 Jun 21;7:13. doi: 10.1186/s13395-017-0130-1 (PMC5480156; doi:10.1186/s13395-017-0130-1)
Supplement: Supplementary file 3 — DUX4 is not expressed in PAX7 positive myocytes in hiPSC-mosaic 1 with the long D4Z4 array. A, B, and C) Images of hiPSC-mosaic 1 long myocytes from D40 of the differentiation protocol stained with antibodies to both PAX7 and DUX4 and utilized to quantify the number of DUX4 and PAX7 positive cells. (DOCX 4880 kb) [file 13395_2017_130_MOESM3_ESM.docx]

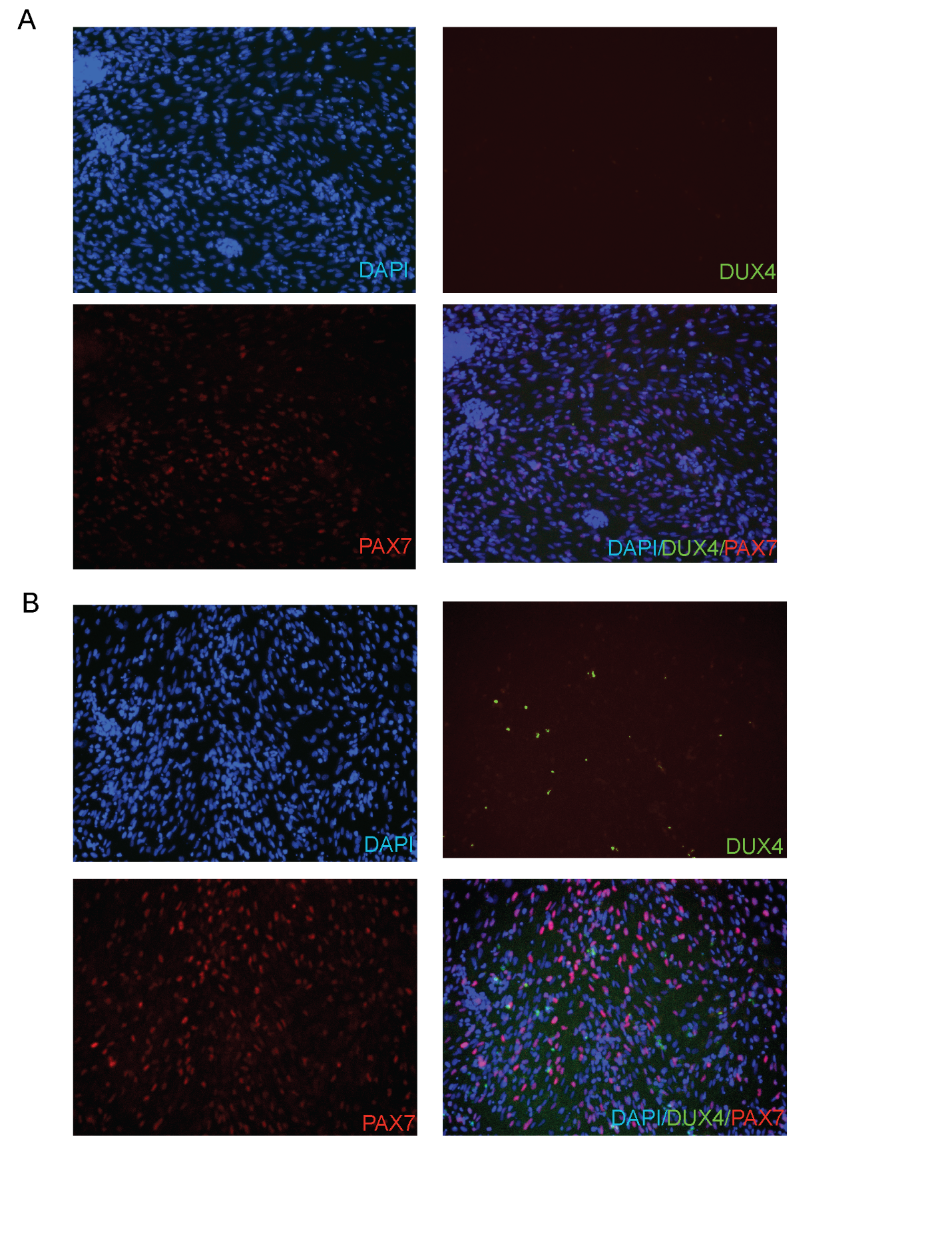


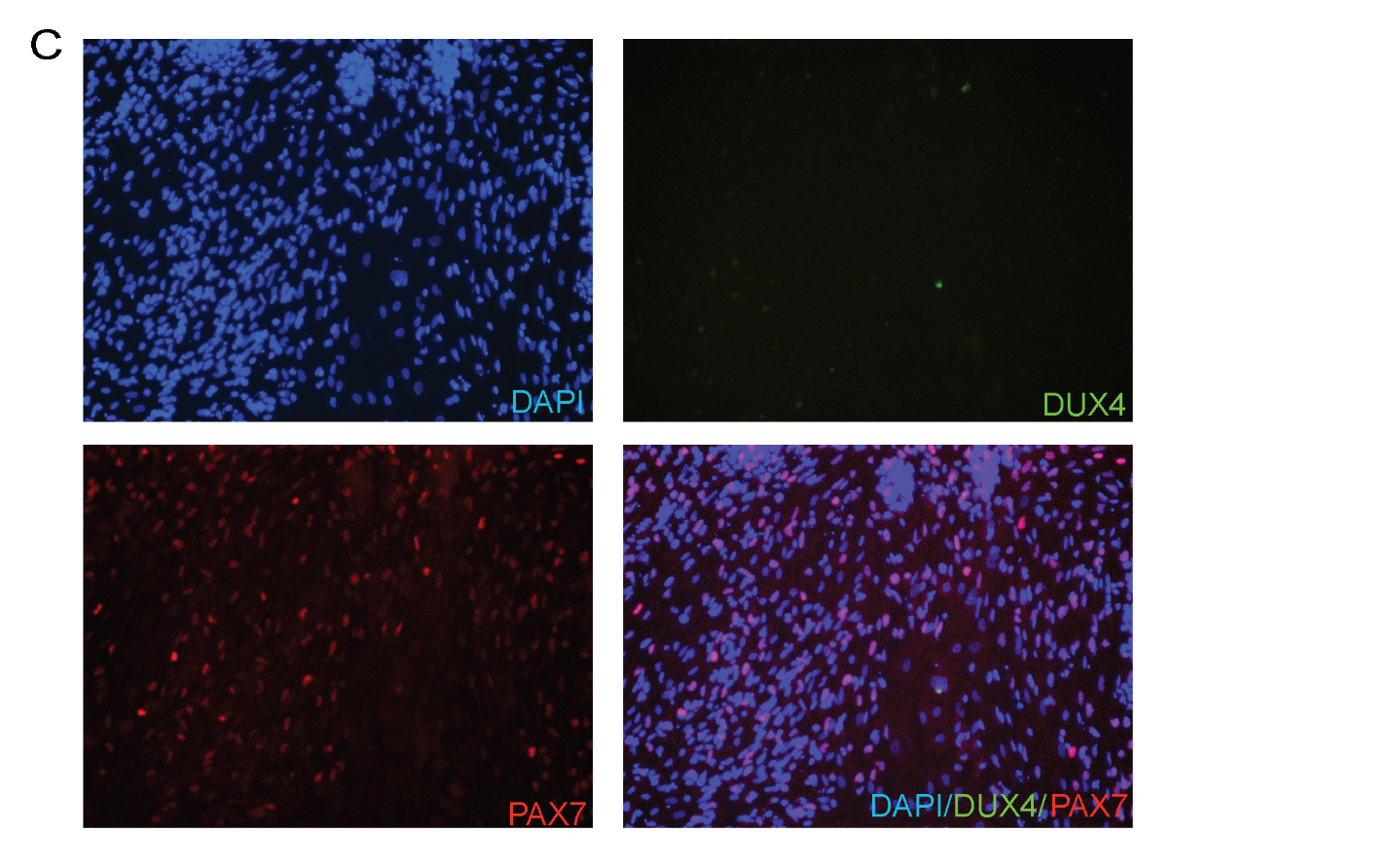


**Additional file 3: Figure S3. DUX4 is not expressed in PAX7 positive myocytes in hiPSC-mosaic 1 with the long D4Z4 array.** A, B, and C) Images of hiPSC myocytes from D40 of the differentiation protocol stained with antibodies to both PAX7 and DUX4 and utilized to quantify the number of DUX4 and PAX7 positive cells.
