# Supplementary material for: Expression patterns of FSHD-causing DUX4 and myogenic transcription factors PAX3 and PAX7 are spatially distinct in differentiating human stem cell cultures
Source: Skelet Muscle. 2017 Jun 21;7:13. doi: 10.1186/s13395-017-0130-1 (PMC5480156; doi:10.1186/s13395-017-0130-1)
Supplement: Supplementary file 4 — DUX4 and PAX7 are expressed in distinct cell types during myogenic differentiation of hiPSC-mosaic 1 with the short D4Z4 array. A, B and C) Images of hiPSC-mosaic 1 with the short D4Z4 array from D40 of the differentiation protocol stained with antibodies to both PAX7 and DUX4. Arrows indicate representative DUX4 positive nuclei counted. (DOCX 5052 kb) [file 13395_2017_130_MOESM4_ESM.docx]

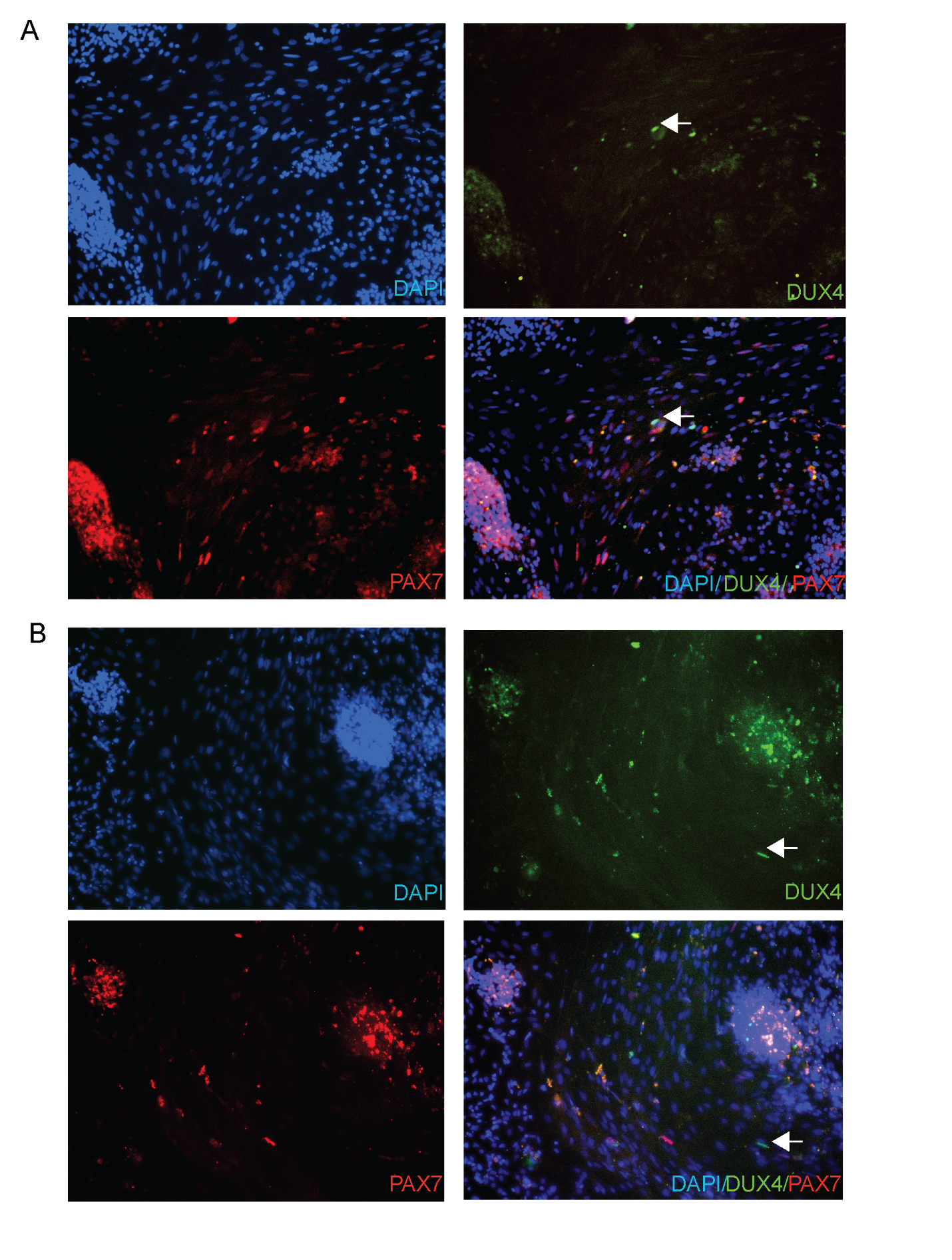


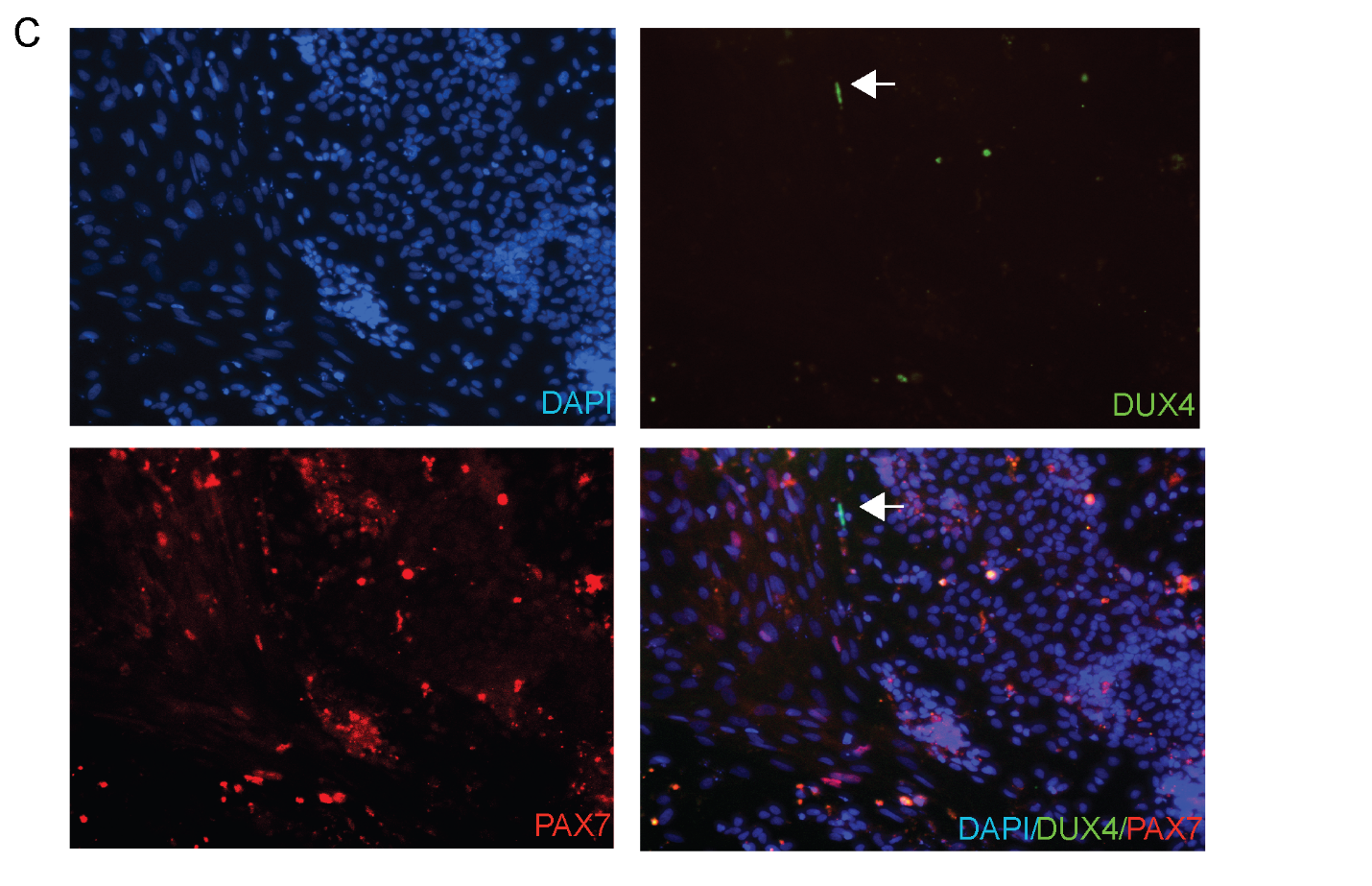


**Additional file 4: Figure S4. DUX4 and PAX7 are expressed in distinct cell types during myogenic differentiation of hiPSC-mosaic 1 with the short D4Z4 array.** A,B and C) Images of hiPSC-mosaic 1 with the short D4Z4 array from D40 of the differentiation protocol stained with antibodies to both PAX7 and DUX4. Arrows indicate representative DUX4 positive nuclei counted.
