# Supplementary material for: Expression patterns of FSHD-causing DUX4 and myogenic transcription factors PAX3 and PAX7 are spatially distinct in differentiating human stem cell cultures
Source: Skelet Muscle. 2017 Jun 21;7:13. doi: 10.1186/s13395-017-0130-1 (PMC5480156; doi:10.1186/s13395-017-0130-1)
Supplement: Supplementary file 8 — DUX4 and PAX7 are expressed in distinct cell types during myogenic differentiation of human ES cells with FSHD. A, B, C, D, E and F) Images of hESC-FSHD from D40 of the differentiation protocol stained with antibodies to both PAX7 and DUX4. Arrows indicate representative DUX4 positive nuclei counted. (DOCX 6109 kb) [file 13395_2017_130_MOESM8_ESM.docx]

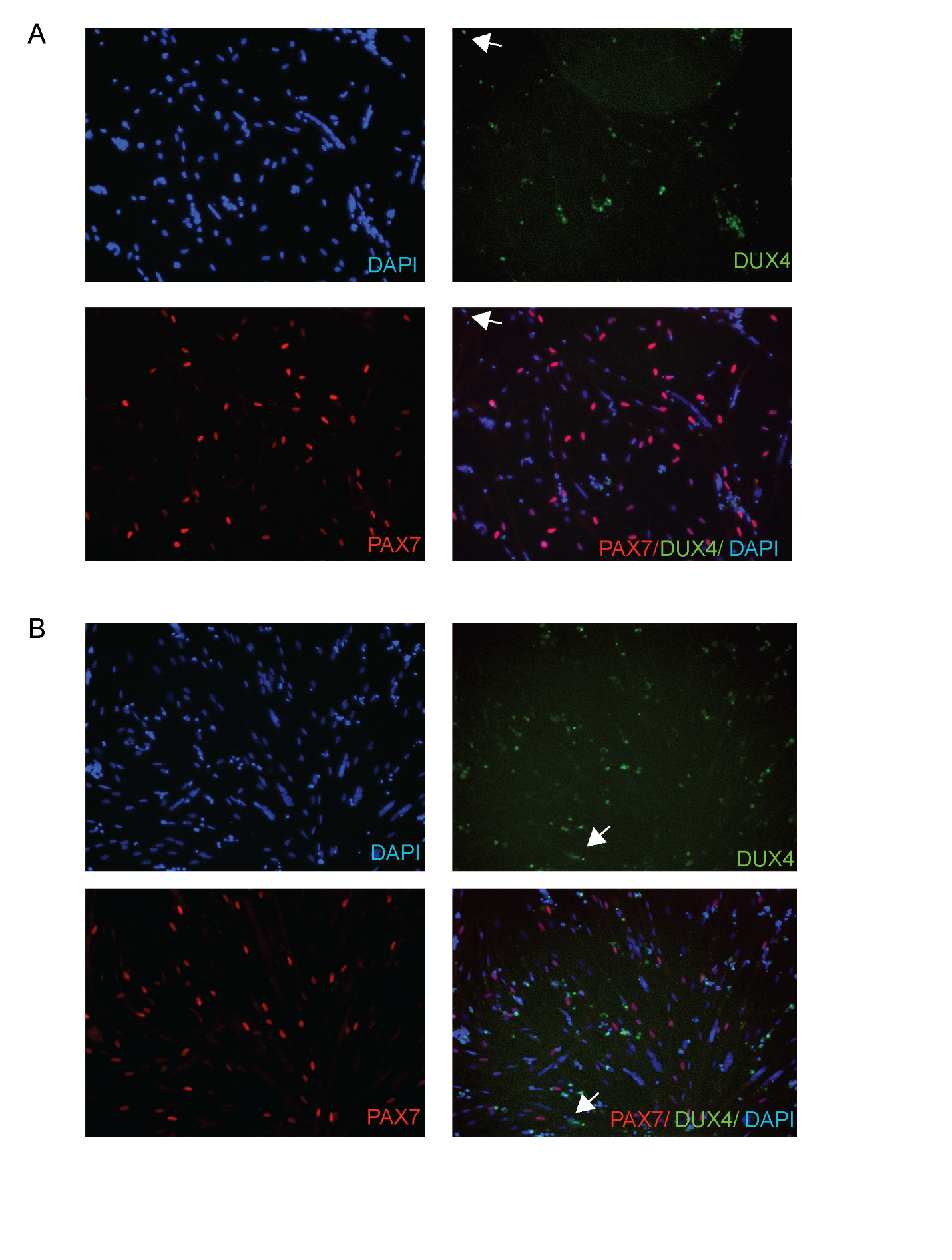


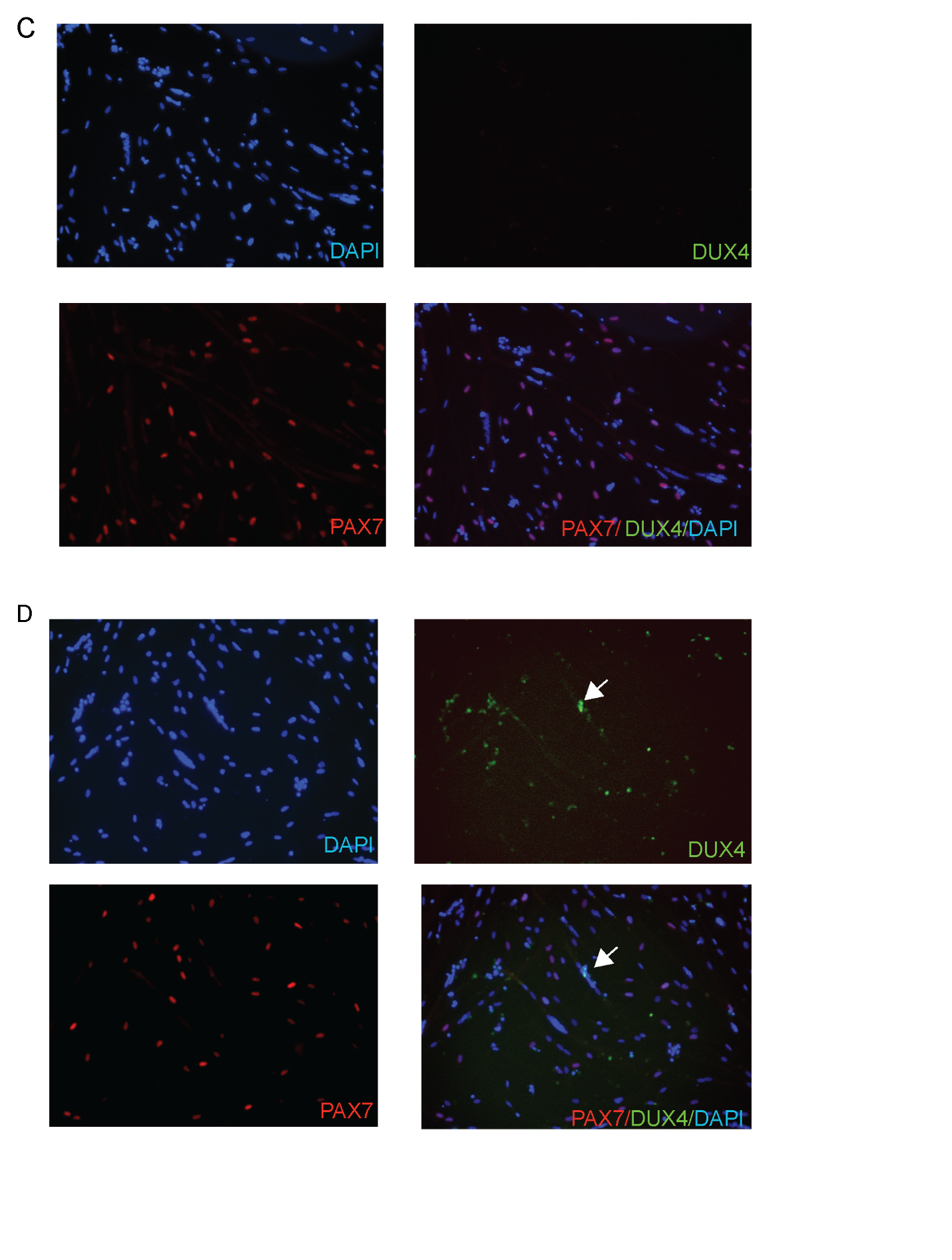


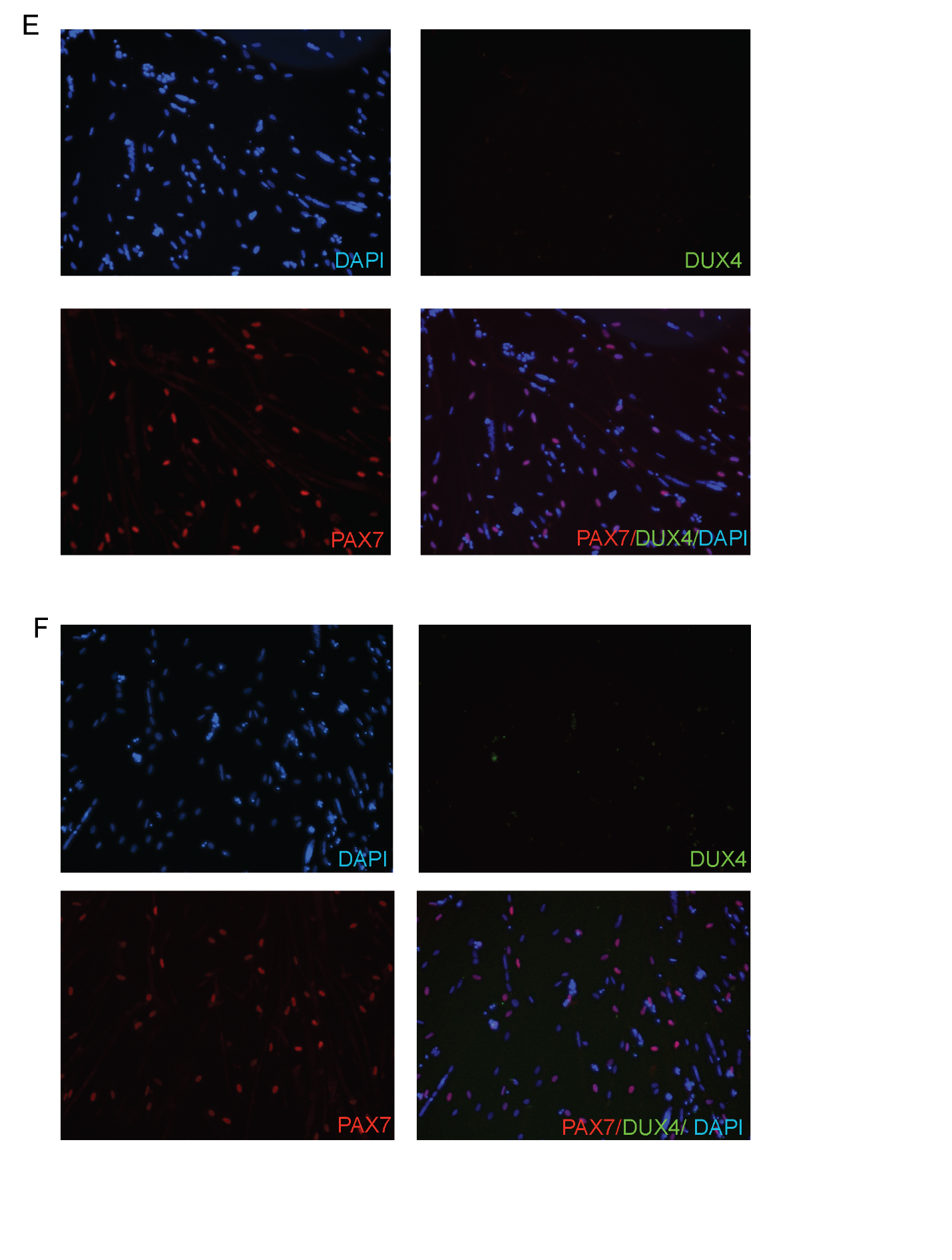


**Additional file 8: Figure S8. DUX4 and PAX7 are expressed in distinct cell types during myogenic differentiation of human ES cells with FSHD.** A, B, C, D, E and F) Images of hESC with FSHD from D40 of the differentiation protocol stained with antibodies to both PAX7 and DUX4. Arrows indicate representative DUX4 positive nuclei counted.
